# Emerging Advances in Endometrial Cancer: Integration of Molecular Classification into Staging for Enhanced Prognostic Accuracy and Implications for Racial Disparities

**DOI:** 10.3390/cancers16061172

**Published:** 2024-03-16

**Authors:** Joy Ogunmuyiwa, Vonetta Williams

**Affiliations:** 1 New York Presbyterian Brooklyn Methodist Hospital, Brooklyn, NY 11215, USA; joo9089@nyp.org; 2 Weill Cornell Medicine Meyer Cancer Center, 1300 York Ave, New York, NY 10065, USA; 3 Memorial Sloan Kettering Cancer Center, New York, NY 10065, USA

**Keywords:** POLE-mutated, mismatch repair deficient, p53 mutant, targeted therapy, staging, endometrial cancer

## Abstract

**Simple Summary:**

This review examines the 2023 FIGO staging update’s integration of molecular and histopathological classifications to improve prognostic accuracy and treatment personalization in endometrial cancer. It highlights the potential of molecular subtyping to address racial disparities in morbidity and mortality, marking a significant shift towards a more nuanced and equitable approach to cancer care.

**Abstract:**

Since the 2009 FIGO staging update, focused exclusively on the anatomic extent of disease, there have been several advances in the understanding of the pathologic and molecular features of endometrial cancer. In a significant departure from the 2009 FIGO staging system, the 2023 FIGO staging update integrates both histopathological and molecular classification. With the inclusion of non-anatomic pathologic parameters such as histology, tumor grade, lymphovascular space invasion, and molecular subtype, the 2023 FIGO staging update aims to create more clinically relevant substages that improve prognostic value and allows for more individualized treatment paradigms. This review will evaluate the clinical impact of the 2023 FIGO staging update, describe the stage shifts that lead to higher prognostic precision, and illustrate the current state of molecular analysis in clinical practice. Furthermore, this review will explore how incorporating factors such as molecular subtype into endometrial cancer staging can offer valuable insights into the racial disparities seen in morbidity and mortality.

## 1. Introduction

With an estimated 66,200 new cases and 13,030 deaths in 2023 alone, endometrial cancer is the most common gynecologic cancer and fourth most common cancer in the United States [1]. Unlike other gynecologic cancers, the incidence and mortality of endometrial cancer is rising [1]. It is expected to surpass colorectal cancer as the third most common cancer and fourth leading cause of cancer death among women in the United States by 2040 [2,3]. This increase in incidence and mortality is seen disproportionately among minority racial groups [1,4,5]. This racial disparity has persisted over the past several decades, and has been attributed to complex, multifactorial issues including higher likelihoods of aggressive disease and histologic subtypes, more advanced stage at presentation, and differences in treatment received [6,7,8,9].

## 2. Current Histopathologic Classification of Endometrial Cancer

Historically, the staging of endometrial cancer, as delineated by the International Federation of Gynecology and Obstetrics (FIGO) guidelines, was determined surgically and primarily focused on the anatomical extent of the disease based on factors such as the involvement of the uterus, cervical extension, involvement of surrounding structures or lymph nodes, and distant spread [10]. The Gynecologic Oncology Group (GOG) surgical manual defines comprehensive surgical staging of endometrial cancer as removal of the uterus, cervix, adnexa, and pelvic and para-aortic lymph node tissues, and the obtainment of pelvic washings [10,11]. The surgical approaches include total hysterectomy and bilateral salpingo-oophorectomy either via open or minimally invasive approaches [12]. Minimally invasive laparoscopic or robotic-assisted techniques are presently the favored methods due to their association with fewer complications and shorter hospital stays [13]. Partial or supracervical hysterectomies are generally not recommended and rarely performed as they are associated with decreased survival among women with even early-stage endometrial cancer [14]. For higher risk endometrial cancers based on factors such as tumor size, >50% myometrial invasion, cervical stromal involvement, histology, or presence of enlarged lymph nodes, a retroperitoneal pelvic and para-aortic lymphadenectomy is performed [15]. Omentectomy, peritoneal biopsies, and peritoneal/abdominopelvic washings are less routinely performed but can be considered in patients with non-endometrioid histology, especially in cases where there is a higher risk of metastatic disease [16].

The surgical staging system was due, in part, to the results of GOG 33 [10,11,17]. GOG 33 was a prospective observational trial from 1977 to 1983 that evaluated 681 patients who underwent total abdominal hysterectomy, bilateral salpingo-oophorectomy, and selective pelvic and para-aortic lymph node dissection [11]. Multivariate analysis demonstrated that tumor grade, depth of myometrial invasion, and intraperitoneal disease were predictive of lymph node metastasis [10]. Based on these findings, three risk categories were defined—low, moderate, and high risk. Low risk disease was characterized by a grade 1 tumor with neither extra-uterine spread nor intraperitoneal disease, and no metastasis to either pelvic or para-aortic lymph nodes [10]. Moderate risk disease was characterized as less than half myometrial invasion without intraperitoneal disease. Those with moderate risk factors had a 3–6% incidence of metastasis to pelvic lymph nodes and a 2% incidence of metastasis to para-aortic lymph nodes [10,11]. High risk disease was characterized by greater than half myometrial invasion and/or intraperitoneal disease. Those with high risk factors were found to have an 18% incidence of metastasis to pelvic lymph nodes and a 15% incidence of metastasis to para-aortic lymph nodes [10,11]. For patients with intraperitoneal disease and only superficial myometrial invasion, the risks of pelvic and para-aortic lymph node metastasis were as high as 33% and 8%, respectively [10,11]. Patients with both high-risk features were at the highest risk, with a 61% and 30% risk of pelvic and para-aortic lymph node involvement, respectively [10,11]. GOG 33 was one of the first clinical trials to demonstrate the benefit of surgical staging by providing evidence that clinical stage 1 disease could include pathological risk factors that warrant adjuvant radiation therapy in up to 15–25% of early-stage patients [10,11]. In addition, upstaging in another 5–9% of patients by extrauterine involvement may occur, significantly affecting prognosis [10,11]. The results of this study prompted a revision of the FIGO staging for endometrial cancer from clinical to surgical staging.

The relationship between increasing FIGO stage and decreasing survival rate has been well established. The Danish Gynecologic Cancer Group (DCCG) population-based studies using the 2009 FIGO staging system demonstrated that from 2005 to 2017, the 5-year survival was 85.1%, 72.2%, 47.0% and 15.6% for stages I–IV, respectively [16]. From 2016 to 2020, the survival rates were 86.3%, 74.6%, 48.7% and 28.2% for 2009 FIGO stages I–IV, respectively [16].

In clinical practice, FIGO staging and histopathologic features are used in conjunction to determine the individual’s risk of recurrence or disease spread and the need for adjuvant treatment in the form of systemic therapy or radiation therapy [18]. 

Several studies have examined the importance of FIGO staging, clinical features, and histopathologic features as prognostic factors for survival and recurrence [11,19,20,21]. These findings guide stratification of patients as low, intermediate, or high-risk for extra-uterine disease or recurrence and in need of adjuvant therapies after surgery. Generally, low risk patients are recommended for observation alone while those with higher risk due to advanced FIGO stage, aggressive histology, high grade, or deep myometrial invasion are advised for more aggressive adjuvant therapies [22]. The importance of adjuvant therapy such as external beam pelvic radiotherapy, vaginal brachytherapy, chemotherapy, and combined chemotherapy and radiotherapy in higher risk patients have been evaluated in several studies such as GOG 258 and PORTEC-3 and have established current standards of care based on histopathology and stage [23,24]. 

However, over the past decade, several groups have demonstrated that certain molecular subtypes of endometrial cancer can have a substantial impact on prognosis, recurrence, and survival outcomes in various cohorts of patients [25]. Molecular analysis and molecular-driven treatments have become an important aspect of patient-directed care in endometrial cancer, offering the potential for more personalized therapeutic strategies [22,26,27,28,29,30]. In addition, clinical trials investigating more personalized therapeutic strategies or different systemic treatment options in patients with advanced/recurrent endometrial carcinoma based on molecular profiles are in progress.

## 3. Molecular Subtypes of Endometrial Cancer

Emerging risk stratification models focused on molecular classification aim at creating tailored treatment algorithms for patients to improve outcomes [31]. The molecular classification of endometrial cancer categorizes endometrial cancers based on distinct molecular and genetic characteristics rather than traditional anatomical or histopathological features alone. The Cancer Genome Atlas (TCGA) research network has been instrumental in advancing this field [32]. Based on comprehensive genomic analysis, TCGA identified four prognostic subtypes of endometrial cancer: DNA polymerase epsilon (POLE) ultra-mutated, microsatellite instability (MSI) hypermutated, copy-number low, and copy-number high [33].

### 3.1. POLE Ultra-Mutated

Characterized by mutations in the exonuclease domain of DNA polymerase epsilon (POLE) impairing the gene’s DNA proofreading function during replication, this subtype is notable for a hypermutator phenotype [30,34]. Next-generation sequencing (NGS) has demonstrated that this high mutational load can predict benefit from immunotherapy due to the immunogenic nature of neoantigens generated from the increased burden of somatic mutations [35,36]. Research indicates that these tumors tend to have an excellent prognosis due to the potent antitumor immune response [33]. Typically, POLE ultra-mutated endometrial cancers are found in younger women with earlier stage, but higher-grade tumors with significant lymphocytic infiltration [37,38,39]. Yet, despite the tendency to be a higher grade, POLE ultra-mutated endometrial cancers offer exceptionally favorable prognosis with only rare recurrence regardless of adjuvant treatment [28,38,40] (Table 1). There is potential for using POLE ultra-mutated status as a marker to de-escalate therapy in certain patient populations [41].

### 3.2. Microsatellite Instability Hypermutated (MSI-H) or Mismatch Repair Deficient (MMRd)

Microsatellite instability hypermutated (MSI-H) tumors exhibit high levels of microsatellite instability due to defects in mismatch repair (MMR) genes [30,35]. The term MSI-H tumors, referring to the phenotype, is often interchanged with the term MMR deficient, referring to the genotype [22]. This group composes approximately 30% of all endometrial cancers and 13–30% of recurrent/metastatic endometrial cancers [42,43]. Substantial lymphovascular invasion (LVSI) is more commonly observed in this group [42,43]. Similar to POLE-mutated cancers, MSI-high endometrial cancers often respond well to immunotherapy, particularly checkpoint inhibitors, due to the high mutational load [37,39]. In the setting of advanced or recurrent endometrial cancer in MMRd patients, two recent Phase III trials, NRG GY018 and RUBY/GOG3031, have demonstrated that there is an unprecedented progression free survival advantage with the addition of immunotherapy (pembrolizumab and dostarlimab, respectively) to the current standard of care systemic therapy, carboplatin and paclitaxel chemotherapy followed by maintenance immunotherapy [25,44,45]. However, their behavior can be unpredictable, and they exhibit an intermediate prognosis, with limited effective systemic therapies [46].

### 3.3. Copy-Number Low

Of the four TGCA molecular subtypes, the most common is the copy-number low subtype [22]. Its molecular characteristics include a relatively low mutational burden, characterized by fewer changes in the copy number variations throughout the genome and fewer deletions or duplications of large sections of DNA [22,47]. This results in more genome stability compared to the “copy-number high” subtype, which exhibits a higher number of these alterations. While the copy-number low subtype has lower mutational burden, they often carry specific mutations in genes that are known to be important in endometrial cancer pathogenesis. The most common mutations affect the PTEN gene, but mutations in other genes like PIK3CA, KRAS, and ARID1A are also frequently observed [47]. Of particular importance is the PI3K pathway, known to play an important role in genomic stability, cell cycle regulation and cell survival, resistance to chemotherapy, and DNA replication [48]. Inhibition of this pathway may lead to genomic instability and mitotic catastrophe [48].

The endometrioid histologic type comprises the majority of the copy-number low category [22]. Copy-number low endometrial cancers often present at an earlier stage and have a favorable prognosis with a lower risk of recurrence [22,47]. This molecular subtype is composed mostly of lower grade tumors with positive estrogen and progesterone receptors when compared with the POLE and MSI-H subtypes, making it generally sensitive to hormonal therapy due to its association with estrogen exposure [22,49]. 

### 3.4. Copy-Number High

The copy-number high subtype is also referred to as “serous-like” and is characterized by high genomic instability and frequent TP53 mutations [22]. This molecular subtype is often aggressive, with poorer prognosis and lower responsiveness to standard therapies [22,28]. Research into targeted therapies, especially those focusing on TP53 pathways and poly-ADP ribose polymerase (PARP) inhibitors which have been found to be successful in the treatment of ovarian and breast cancer, is active for this subtype [22,50]. In addition to ovarian and breast cancer, germline or somatic aberrations in DNA damage repair genes are present in nearly 20% of primary prostate cancers and nearly a quarter of metastatic castration-resistant prostate cancer [51]. Consequently, various PARP inhibitors have been explored for use in patients with metastatic castration-resistant prostate cancer and have shown effectiveness in those with germline BRCA2 mutations [51]. 

## 4. Re-Classification of TCGA Subtypes for Clinical Use

To simplify the TCGA classification for everyday clinical use, methods that align with the TCGA classification system but are both reliable and cost-efficient have been suggested [28,52]. One such system is the Proactive Molecular Risk Classifier for Endometrial Cancer (ProMisE). ProMisE defines four molecular subtypes that mirror the four molecular categories outlined by TCGA: POLE, MMRd, No specific molecular profile (NSMP) representing copy number low, and p53 abnormal (p53abn) representing copy number high [52] (Table 2). This classification relies on straightforward, cost-effective molecular assays that are routinely employed in clinical settings: genetic sequencing to identify mutations in the POLE exonuclease domain, MMR immunohistochemistry (IHC) for MLH1, MSH2, MSH6, and PMS2 to detect MMR deficiency, and IHC for p53 (distinguishing between wild-type, p53wt, and mutant-type expression, p53abn) [32]. These methods can be used in standard formalin-fixed paraffin-embedded material [32]. Although the ProMisE system offers a streamlined approach to molecular classification, it is important to acknowledge that variations in IHC testing results for MMR can arise due to somatic mutations, underscoring the need for ongoing research to refine diagnostic accuracy and therapeutic stratification in light of biological and technical heterogeneity [53].

### 4.1. p53 Abnormal (p53abn)

The p53 abnormal subtype is characterized by deleterious mutations in the TP53 gene, which encodes the tumor protein p53, an important regulator in many cellular processes, including DNA repair, cell cycle, apoptosis, and senescence. The p53 abnormal subtype corresponds to the copy-number high group [52]. While they are found often in the copy-number high category, they can be present in other subtypes as well. p53 abnormal endometrial cancers are often associated with worse prognosis, including a higher risk of recurrence and reduced survival [28,33]. These tumors tend to be more aggressive, likely due to the p53 protein’s central role in regulating cell cycle checkpoints and apoptosis [32,36]. The p53 abnormal subtype poses significant therapeutic challenges as the standard of care therapy, surgery and chemotherapy, is often less effective in this subtype due to the tumors’ aggressive nature and potential for resistance to therapy [54].

### 4.2. No Specific Molecular Profile (NSMP)

While the No specific molecular profile (NSMP) molecular subtype generally corresponds to the copy-number low group of the TGCA classification, tumors in this category do not fit as clearly into these molecular subtypes. They lack the high mutation burden of POLE and MSI-high cancers and do not exhibit the marked copy-number alterations or specific mutations of the other categories [32]. They have preserved p53 and MMR immunohistochemical expression, corresponding to the copy-number low group, having a low mutational burden [32]. There is limited literature on this subtype, but its identification suggests a heterogeneity within endometrial cancer that requires further research for tailored therapeutic approaches [42].

### 4.3. Evidence for the Prognostic Significance of the Molecular Subtypes

The prognostic significance of molecular classification was examined in a retrospective, combined analysis of the PORTEC-1 and PORTEC-2 cohorts, two key clinical trials that have shaped the management of endometrial cancer [55]. In this analysis, historical patient tissue samples of 880 women with high-intermediate risk endometrial cancer were re-evaluated and categorized into the relevant TCGA subgroups: POLE ultra-mutated, microsatellite instability (MSI) hypermutated, copy-number low, and copy-number high. Molecular analysis was feasible in >96% of the patients. This enabled a direct comparison between the molecular classification and the traditional clinicopathological risk factors, focusing on their ability to predict disease recurrence, progression, and patient survival. They found that there were no locoregional recurrences in POLE-mutant subtypes regardless of the presence or absence of adjuvant radiotherapy. They also found that within the MMRd subtypes, there was no significant difference in locoregional recurrence-free survival after external beam radiation therapy, vaginal brachytherapy, or no adjuvant therapy. However, within the p53-abnormal subtype, there was significantly better locoregional recurrence-free survival in the external beam radiation therapy group compared to vaginal brachytherapy group or no adjuvant therapy group. For women with the NSMP subtype, there was significantly better locoregional recurrence-free survival with both external beam radiation therapy and vaginal brachytherapy when compared with no adjuvant therapy. The study underscored the potential of molecular classification to serve as a valuable tool in predicting the response to radiotherapy among patients with early-stage endometrioid endometrial cancer, promoting more personalized and effective treatment strategies.

Building on this, the prognostic capability of molecular classification was further investigated in the high-risk patient cohort of the PORTEC-3 trial. PORTEC-3 sought to investigate the benefit of chemoradiation versus pelvic radiotherapy alone for women with high-risk endometrial cancer. Once again, a retrospective analysis using tissue samples of 410 women from the PORTEC-3 clinical trial, was performed to ascertain whether the molecular subtypes, which had shown prognostic significance in the earlier trials, could also predict responses to the more intensive treatment regimens used in PORTEC-3 [40]. Molecular analysis was feasible in >97% of the patients. Similar to the PORTEC-1 and PORTEC-2 cohorts, molecular subtypes in the PORTEC-3 trial were predictive of outcomes. They found there was significantly improved recurrence free survival with adjuvant chemoradiation for p53-abnormal subtypes, regardless of histologic type. They also found that women with the POLE-mutant subtypes had excellent recurrence free survival regardless of intervention, chemoradiation or radiation alone. They were able to conclude patients with p53-abnormal subtypes should be considered for adjuvant chemoradiotherapy, whereas for those with the POLE-mutant subtype, de-escalation of adjuvant treatment could be considered [40].

Overall, the retrospective analysis of the three PORTEC cohorts demonstrated that molecular classification provides valuable prognostic information that can complement the traditional histopathological classification. These insights suggest that molecular classification could potentially guide individualized treatment plans, and inform more nuanced risk stratification, potentially leading to more personalized, effective treatment approaches. To evaluate the role of a molecular-integrated risk profile in the selection of adjuvant treatment in patients with higher risk endometrial cancer, PORTEC 4a, a prospective, multicenter, randomized phase III trial was opened in 2016 [56]. The trial investigates the role of a molecular-integrated risk profile among women with high-intermediate risk features in determining if participants should receive no adjuvant therapy, vaginal brachytherapy or external beam radiotherapy as compared to standard adjuvant vaginal brachytherapy alone. This is the first randomized trial using molecular risk factors to assign adjuvant treatment for women with stage I–II (FIGO 2009 staging) high-intermediate risk endometrial cancer [56].

## 5. 2023 FIGO Staging Update

Over the course of its decade usage, the FIGO 2009 staging system demonstrated several notable shortcomings including inadequate consideration of the histological type, disregard of lymphovascular space invasion, absence of distinctions based on nodal metastasis size, and lack of molecular subtype classification [36]. However, since the last update of the FIGO staging system in 2009, there have been profound advances in the understanding of endometrial carcinoma. In October of 2021, the FIGO Women’s Cancer Committee established the Endometrial Cancer Staging subcommittee to update the 2009 FIGO staging system, with the aim of creating more detailed subclassifications and better prognostic groups for more appropriate therapeutic approaches [25]. They also adopted risk stratification methodologies developed by organizations like the European Society of Gynaecological Oncology (ESGO), the European Society for Therapeutic Radiology and Oncology (ESTRO), and the European Society of Pathology (ESP).

### 5.1. Lymphovascular Space Invasion

Numerous studies have underscored the prognostic significance of LVSI in endometrial cancer. It is associated with a higher risk of lymph node metastases and recurrent disease [57]. In clinical practice, it plays a large role in guiding adjuvant therapy decisions. In the past, the identification of LVSI, independent of anatomical extent, was a key consideration for proposing adjuvant radiation therapy in stage I, grade 1 or 2 endometrioid endometrial cancer patients [18]. With the introduction of the revised FIGO 2023 staging system, LVSI, which was formerly excluded from staging considerations, is now acknowledged as a critical factor in defining stage II endometrial cancer. This modification within the FIGO 2023 framework highlights the increased prognostic significance given to tumors with extensive LVSI, particularly when found in what would otherwise be classified as stage I disease [36]. However, the FIGO 2023 update does not clarify whether the extent of LVSI is based on the maximum involvement in a single tissue section or on the cumulative extent across all tissue sections [58]. It should be noted that this oversight calls into question the reproducibility of LVSI quantification across institutions, leading to potential difficulties in comparability between practices and regions and potentially inappropriate upstaging of patients [58].

### 5.2. Lymph Node Metastasis

Despite the evidence demonstrating the diagnostic value of pelvic lymphadenectomy, the routine use in managing endometrial cancer has been contested. The debate stems from a lack of randomized trial-based evidence of clinically significant therapeutic benefits, increased morbidity, and no significant improvements in overall or recurrence-free survival rates [59,60]. Consequently, clinicians have gravitated towards a minimally invasive alternative—sentinel lymph node (SLN) mapping, which offers high sensitivity, specificity, and excellent negative predictive values [59]. When compared to pelvic lymphadenectomy, SLN mapping with ultrastaging may increase the detection of lymph node metastasis with low false-negative rates in patients with uterine-confined disease [18,61,62,63]. SLN mapping can be considered for the surgical staging of uterine-confined malignancy when there is no metastasis demonstrated by imaging studies or no obvious extrauterine disease at exploration [18]. SLN mapping decreases morbidity, notably in the incidence of lymphedema and lymphocele, compared to comprehensive lymphadenectomy, while improving the identification of nodal metastases [64]. Thus, the ESGO-ESTRO-ESP guidelines allow SLN in all patients with endometrial carcinoma, which was reaffirmed by the FIGO 2023 restaging. However, a SLN biopsy should be performed in association with ultrastaging to ensure detection of low volume metastasis (LVM). LVM encompasses isolated tumor cells (ITCs) and micrometastases. Micrometastasis is defined as 0.2–2 mm in size and/or >200 cells, and isolated tumor cells are defined as ≥0.2 mm in size and ≤200 cells [25]. LVM accounts for an estimated 8% rise in nodal positivity beyond conventional pathological staging and correlates with a more favorable prognosis compared to macrometastases, defined as >2 mm in size [25].

In the 2023 FIGO staging, there is now a staging distinction between micrometastasis and macrometastasis. While stage IIIC1 and stage IIIC2 are still defined as metastasis to the pelvic lymph nodes and para-aortic lymph nodes, respectively, these stages are further distinguished by micrometastasis (i) and macrometastasis (ii). For example, stage IIIC1i now denotes micrometastatic pelvic lymph node disease, while stage IIIC1ii denotes macrometastatic pelvic lymph node disease.

While ITC is associated with a better prognosis than macrometastasis, the overall prognostic significance of ITC still remains unclear [65]. Clinically, ITCs and micrometastases do not present considerable variances in recurrence and survival outcomes, which validates the omission of ITCs from the revised staging schema [25,36,66].

## 6. 2023 Update to Histology

Central to the 2023 FIGO restaging is once again histopathology, but now with a distinction based on degree of aggressiveness. The updated system classifies endometrial cancers into two main groups: non-aggressive and aggressive [25,36]. Several distinct histological types are acknowledged, (1) endometrioid carcinoma, further divided into low grade (grades 1 and 2) and high grade (grade 3); (2) serous carcinoma (SC); (3) clear cell carcinoma (CCC); (4) mixed carcinoma (MC); (5) undifferentiated carcinoma (UC); (6) carcinosarcoma (CS); (7) other rare forms, including mesonephric-like; and (8) gastrointestinal mucinous carcinomas [25] . The non-aggressive histology group includes endometrioid endometrial carcinomas (EECs) of low-grade or FIGO grade 1–2 [25]. The aggressive group encompasses all high-grade endometrial cancers, including FIGO grade 3 EEC and all non-endometrioid endometrial carcinomas (NEECs) [25]. Now, for example, for disease confined to the uterine corpus and ovary (stage I), presence of an aggressive histological type upstages a patient to stage IC regardless of depth of myometrial invasion [25].

However, it is important to recognize that not every grade 3 endometrioid endometrial carcinoma inherently presents with aggressive behavior. This group is heterogeneous both in behavior, molecular classification and prognosis. As discussed earlier, this group benefits dramatically from applying molecular classification, effectively differentiating between those with an exceptionally favorable prognosis (POLE mutations) and those with a poor prognosis (p53 abnormal). For example, recent studies have indicated that grade 3 endometrioid endometrial carcinomas with a NSMP, particularly those negative for estrogen receptor (ER), tend to also have an unfavorable prognosis [67]. Consequently, without employing molecular classification, high-grade endometrioid endometrial carcinomas cannot be accurately assigned to a risk category.

## 7. Molecular Subtype

Based on a comprehensive analysis of the existing data, FIGO now advocates for the integration of molecular subtype evaluation into endometrial carcinoma staging criteria where possible, enhancing the accuracy of prognostic predictions within these frameworks. With the 2023 restaging, there is a strong emphasis on performing molecular subtype evaluation, including POLEmut, MMRd, NSMP, and p53abn, for every case of endometrial carcinoma. This practice is crucial for precise prognostic risk categorization and may also influence decision making in adjuvant therapies. They note that molecular subtyping can be performed on the biopsy sample rather than on the hysterectomy specimen, which may allow for better performance of immunohistochemical and molecular techniques [25].

For early stages of endometrial cancer, the identification of POLE mutations or p53 abnormal subtypes now plays a pivotal role in determining the FIGO stages. Specifically, for tumors initially classified as stage I or II, based on purely surgical, anatomical, and histological parameters, the 2023 staging stipulates that a POLEmut subtype, irrespective of cervical involvement, LVSI status, or histological type, is reassigned as stage IAmPOLEmut [25]. Conversely, a p53abn subtype, irrespective of cervical involvement, myometrial invasion, LVSI status, or histological type, is reclassified as stage IICmp53abn [25]. It is also noteworthy that, despite limited data, in rare cases where low grade endometrioid endometrial cancer restricted to the uterus demonstrates the p53 abnormal subtype, it is upstaged to stage IIC2mp53abn [25]. In scenarios where the tumor demonstrates POLEmut or MMRd with a secondary p53 anomaly, the classification prioritizes the POLEmut or MMRd status for staging purposes [25].

## 8. Verification of the New Staging System

There is emerging evidence that the 2023 FIGO staging system has better prognostic precision compared to the 2009 FIGO staging system, particularly with the substages in early stages of the disease adding further prognostic granularity. A pooled retrospective analysis of 519 patients assessed the stage transitions between the 2009 and 2023 FIGO staging systems, along with the prognostic accuracy of the 2023 FIGO system [68]. The analysis demonstrated a significant stage shift in over a quarter of the patients, leading to improved prognostic precision [68]. Furthermore, the analysis found that the 2023 FIGO system was better able to predict progression-free survival than the 2009 system [68].

## 9. Racial Disparities in Endometrial Cancer

Substantial racial disparities in the incidence, treatment and survival outcomes of women with endometrial cancer are well established [69,70,71]. These disparities are most starkly observed between non-Hispanic white women and African American women. Until recently, while non-Hispanic white women have a higher incidence of endometrial cancer, African American women fared worse survival outcomes, even after adjusting for factors like age, FIGO stage, and histologic subtype [69]. However, recent data indicates that now both incidence and mortality are increased in African American women [1]. The estimated 5-year relative survival (all stages and subtypes included) in 2018 was 84% for white women and only 62% for African American women [72].

Several reasons have been identified as to why the burden of endometrial cancer is not equally distributed across racial and ethnic groups. This discrepancy is often attributed to a higher prevalence of aggressive histologic tumor subtypes, advanced stage at diagnosis, and increased prevalence of comorbidities among African American women [69,73]. The more aggressive serous and clear cell histologic subtypes of endometrial cancer are more prevalent among African American women, while the less aggressive endometrioid subtype are found to be more common in non-Hispanic white women [71]. African American women are less likely to have POLE ultra-mutated cancers, which tend to have a better prognosis [74]. They are also more likely to harbor the TP53 mutation, associated with the more aggressive endometrial serous carcinoma and poorer outcomes [74,75]. Conversely, PTEN mutations, more commonly seen in the less aggressive endometrioid endometrial carcinoma, are more prevalent in NHW women [74,75]. HER2 neu oncogene expression, associated with increased treatment resistance and consequently poorer survival, has been found to have increased receptor expression in African American women with endometrial serous carcinoma (70%) compared to non-Hispanic white women (24%) [8,76].

The literature clearly demonstrates the existence of racial disparities in treatment and survival outcomes of endometrial cancer. Identified factors that contribute to theses persistent disparities include advanced stage at diagnosis, lower response rates to chemotherapy, the prevalence of high-risk histology and more aggressive molecular subtypes, and racially patterned external environments (e.g., discrimination, poverty) on physiologic and molecular biology [70]. However, there remains an ongoing need for more research to understand the complex interplay of biological, genetic, and socioeconomic factors that contribute to this disparity. Significant knowledge gaps in understanding these disparities in endometrial cancer have been identified, including intervention studies to address racial disparities in guideline-concordant care and the role of health care system access in early detection [66,73]. There is also a notable lack of qualitative research aimed at evaluating the perspectives of minority women who have been diagnosed with endometrial cancer [66,73].

Though restaging based on molecular subtypes alone may not fully explain all the disparities, it can provide valuable insights into the disease, better predicting clinical outcome in patients and potentially contributing to a better understanding of racial disparities in endometrial cancer morbidity and mortality.

## 10. Conclusions

While FIGO staging and histopathologic features remain the cornerstone of endometrial cancer staging and treatment, its limitations are evident in the varied clinical outcomes of patients with similar histopathological features, especially among those with high-grade cancers. The revisions in the 2023 FIGO staging and a new emphasis on molecular directed treatment reflect an evolution in understanding, heralding a more personalized era of patient management. This more targeted approach is not only pivotal for individualized patient care but can aid in addressing broader health inequities and guiding future research, policy, and clinical endeavors in the realm of endometrial cancer.

## Figures and Tables

**Table 1:** Prognostic risk-group stratification.

Prognosis	Molecular Subtype
Good	POLE mutation (POLEmut)
Intermediate	Mismatch repair deficiency (MMRd)/microsatellite instability and no specific molecular profile (NSMP)
Poor	p53 abnormal (p53abn)

**Table 2:** Re-classification of TCGA Subtypes for Clinical Use.

TCGA Prognostic Molecular Subtype	ProMisE Re-Classification of TCGA Subtype	Diagnostic Test
POLE Ultra-Mutated	POLE	Genetic sequencing to identify mutations in the POLE exonuclease domain
Microsatellite Instability Hypermutated (MSI-H) or Mismatch Repair Deficient (MMRd)	MMRd	MMR IHC for MLH1, MSH2, MSH6, and PMS2 to detect MMR deficiency
Copy-Number Low	No specific molecular profile (NSMP)	NA
Copy-Number High	p53 abnormal (p53abn)	IHC for p53 (distinguishing between wild-type, p53wt, and mutant-type expression, p53abn)

## Data Availability

No new data were created or analyzed in this study. Data sharing is not applicable to this article.
